# Electron transfer in multicentre redox proteins: from fundamentals to extracellular electron transfer

**DOI:** 10.1042/BSR20240576

**Published:** 2025-01-30

**Authors:** Büşra Bayar, Ricardo Soares, Haris Nalakath, Alexandra Alves, Catarina M. Paquete, Ricardo O. Louro

**Affiliations:** 1 Instituto de Tecnologia Química e Biológica António Xavier, Universidade Nova de Lisboa, Oeiras, Portugal; 2 Instituto Nacional de Investigação Agrária e Veterinária, Oeiras, Portugal

## Abstract

Multicentre redox proteins participate in diverse metabolic processes, such as redox shuttling, multielectron catalysis, or long-distance electron conduction. The detail in which these processes can be analysed depends on the capacity of experimental methods to discriminate the multiple microstates that can be populated while the protein changes from the fully reduced to the fully oxidized state. The population of each state depends on the redox potential of the individual centres and on the magnitude of the interactions between the individual redox centres and their neighbours. It also depends on the interactions with binding sites for other ligands, such as protons, giving origin to the redox-Bohr effect. Modelling strategies that match the capacity of experimental methods to discriminate the contributions of individual centres are presented. These models provide thermodynamic and kinetic characterization of multicentre redox proteins. The current state of the art in the characterization of multicentre redox proteins is illustrated using the case of multiheme cytochromes involved in the process of extracellular electron transfer. In this new frontier of biological electron transfer, which can extend over distances that exceed the size of the individual multicentre redox proteins by orders of magnitude, current experimental data are still unable, in most cases, to provide discrimination between incoherent conduction by heme orbitals and coherent band conduction.

## Introduction

Taking the cue from the Nobel Prize winner in Physiology or Medicine, Albert Szant-Györgyi, who stated that ‘Life is nothing but an electron looking for a place to rest’, this paper focuses on the processes of transfer of multiple electrons in biological systems. It examines the strategies to determine the thermodynamic and kinetic properties of redox proteins and how redox processes are choreographed with the transfer of other charges, such as protons, and the implications of this choreography for the conductivity of structures made of redox-active biomolecules. This process is illustrated using the case of extracellular electron transfer (EET). This metabolic process links the bioenergetic metabolism of some bacteria and archaea with electron donors or acceptors that do not permeate the cell envelope. These can be minerals in the environment or electrodes in devices that extract or provide electricity to sustain microbial metabolism and perform useful work. EET holds great promise for developing novel and sustainable industrial applications. These include sustainable energy production from waste streams [1], water desalination [2], biosensing [3], production of added-value compounds [4], bioelectronics [5], and biomaterials [6].

### Thermodynamics of multielectron transfer in biological systems

The reduction potential of a single redox centre can be determined by the application of the Nernst equation:


(1) $$E=E^{0}-\frac{RT}{nF}ln\frac{\left[red\right]}{\left[ox\right]},$$

­where *E* is the solution potential, *E*^0^ is the potential of the redox centre in standard conditions, *R* is the gas constant, *T* is the absolute temperature, *n* is the number of electrons exchanged in the process, and *F* is the Faraday constant. Plotting [red] or [ox] vs. *E* generates a redox titration curve. This equation applies, for example, to the case of mitochondrial cytochromes *c* or high-potential iron–sulfur proteins (HiPIPs) [7,8].

**Figure 1:**
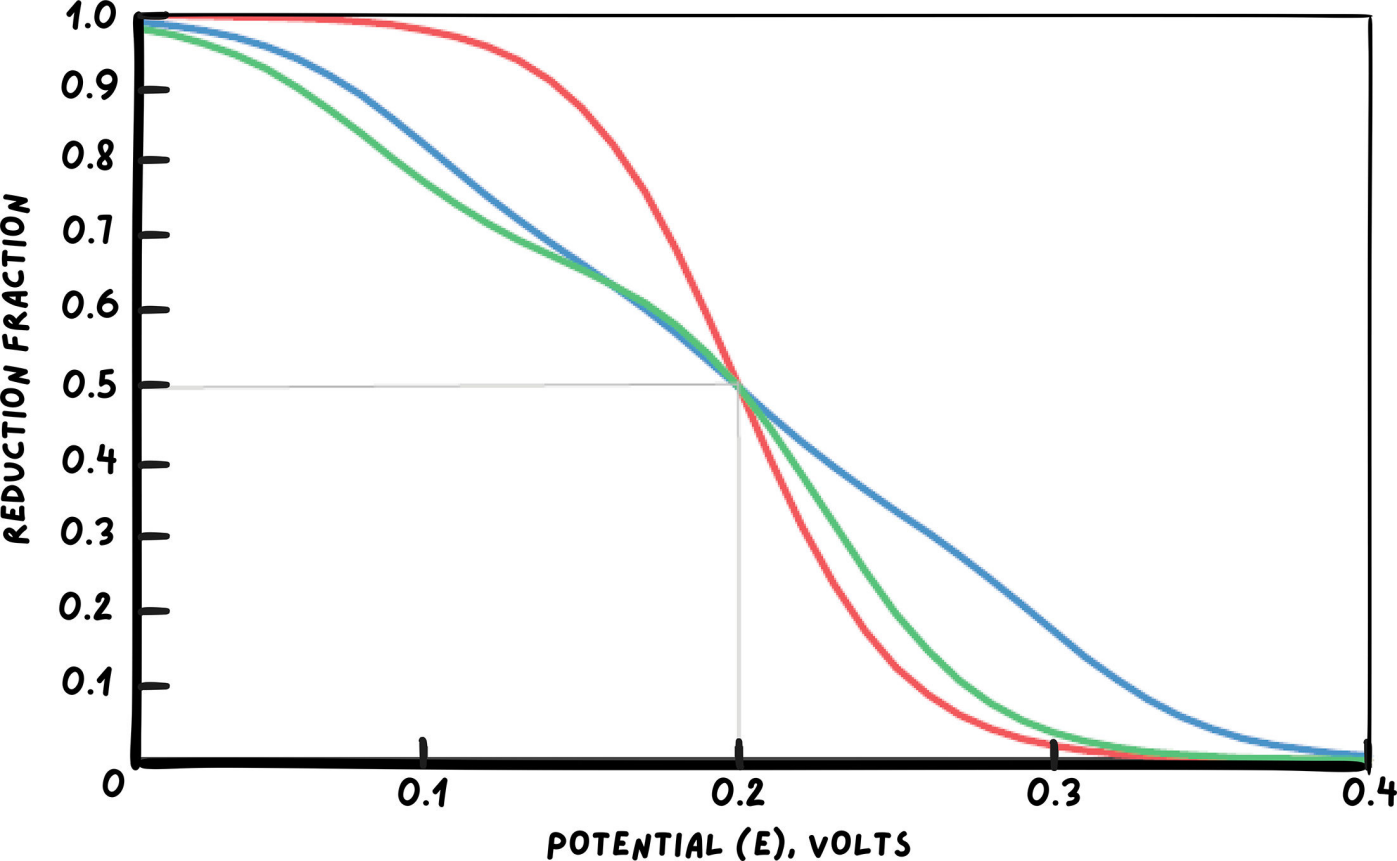
Illustration of three scenarios for proteins with the same apparent mid-point potential (200 mV), but where the distribution of the potentials of the individual independent *n* = 1 redox centres is different. The curves were calculated considering the sum of contributions from eqn 1. The curve in red was calculated considering that all centres have a potential E_0_ of 200 mV; the curve in blue was calculated considering that all centres have different potentials E_0_ of 100, 200, and 300 mV; and the curve in green was calculated considering that two centres have a potential E_0_ of 227 mV and one has a potential E_0_ of 83 mV.

When the protein contains more than one redox centre, the characterization of its redox properties becomes more complex. On the one hand, the shape of the overall redox titration curve depends on the actual distribution of the potentials of the redox centres (Figure 1). Therefore, reporting an apparent midpoint potential for the protein (the potential where the reduced fraction is 50%) provides very little meaningful functional information, as illustrated in Figure 1 for the simple case of a three-centre protein. In these conditions, it is more relevant to report the redox active potential window [9].

**Figure 2:**
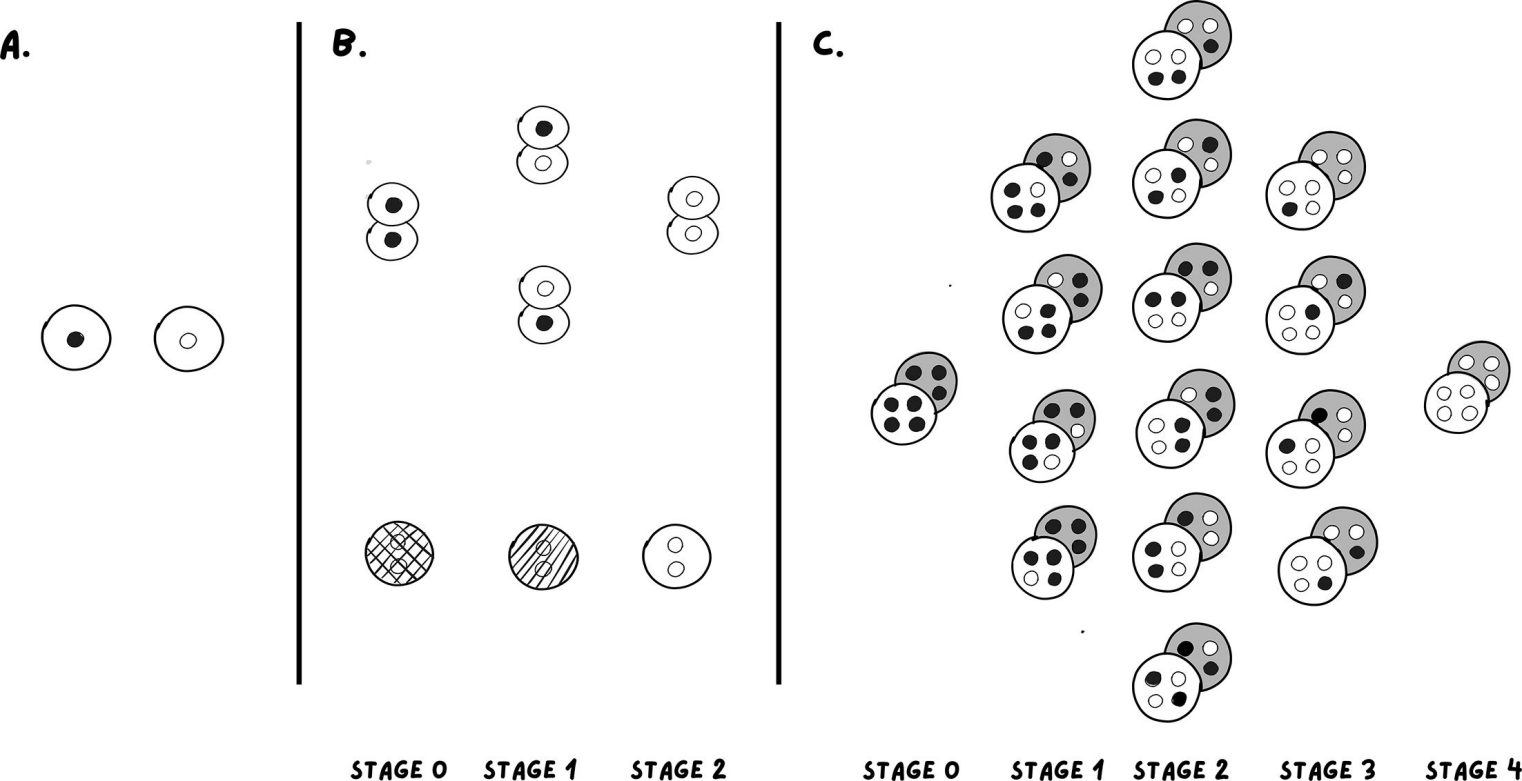
Diagram illustrating the total microstates to be considered for a protein with one redox centre (**A**), two redox centres (**B**), four redox centres and an acid–base centre (**C**). Black and white dots represent reduced and oxidized redox centres, respectively. White and grey circles represent deprotonated and protonated microstates, respectively. Crossed and striped circles represent the fully reduced and the semi-reduced protein.

On the other hand, the level of detail of the analysis that can be performed depends on the capacity to discriminate the contributions of the individual redox centres to the overall protein redox properties, as illustrated in Figure 2.

When the experimental data do not discriminate the contributions of individual centres, the overall redox process can be described by a sum of *j* Nernst equations. Here, *j* is the total number of electrons that the protein can take up or release when transitioning from the fully oxidized to the fully reduced state,


(2) $$E=\sum_{j}E_{j}^{0}-\frac{RT}{nF}ln\frac{{\left[red\right]}_{j}}{{\left[ox\right]}_{j}},$$

This is the simplest analysis that determines the redox potentials of individual centres that can be performed in the absence of structural information on the protein. However, this analysis is framed by the implicit assumption that the redox centres are *independent* and *non-interacting* (Figure 2B; top). In nature, in the relatively low dielectric medium inside proteins, this assumption is only valid for relatively long distances between the redox centres. For example, in multiheme cytochromes (MHCs) *c*, redox interactions between neighbouring hemes follow a damped Coulomb distance dependence where their magnitude is reduced below 5 mV only beyond 17 Å [10]. This contrasts with the observed distribution of distances between redox centres in multicentre proteins, where distances between neighbouring centres are clustered below 15 Å [11]. Given these circumstances, when it is known that the distance between the redox centres is not compatible with the assumption of independent titration, an alternative approach can be used to characterize a multicentre redox protein. This approach describes the sequential uptake or release of electrons from the redox protein. These potentials are not potentials of individual centres but instead *macroscopic potentials* for the *ordered sequential* uptake or release of electrons by the protein (Figure 2B; bottom). This approach can also be used when there are no experimental handles to discriminate the individual redox centres. Without loss of generality, the equations for the case of a two-centre protein are


(3) $$E_{1}=E_{1}^{0}-\frac{RT}{nF}ln\frac{\left[semi\right]}{\left[ox\right]},$$

(4) $$E_{2}=E_{2}^{0}-\frac{RT}{nF}ln\frac{\left[red\right]}{\left[semi\right]},$$

where semi is the sum of the populations with one centre reduced.

In this case, the total fraction of reduction in the protein is given by


(5) $$\frac{2\left[red\right]+\left[semi\right]}{2\left(\left[red\right]+\left[semi\right]+\left[ox\right]\right)}.$$

In eqns 3-5, [ox], [semi] and [red] are the consecutive macroscopic stages of reduction of the protein from fully oxidized to fully reduced. These can be arbitrarily numbered: stage 2 (ox), stage 1 (semi) and stage 0 (red) (Figure 2B).

These two approaches, independent redox centres and sequential redox transitions, provide the same number of potentials from the experimental data. However, in the former, they are *individual potentials* of *non-interacting centres*, whereas in the latter, they are *macroscopic sequential potentials* of the *protein*. For this reason, the values have a different physical meaning and cannot be compared.

When the multiple centres in a redox protein can be experimentally distinguished so that their redox status can be monitored individually, a more detailed analysis can be made to discriminate the individual microscopic redox states of the protein. This *microscopic* analysis defines individual potentials for the centres as well as their interactions.

**Figure 3:**
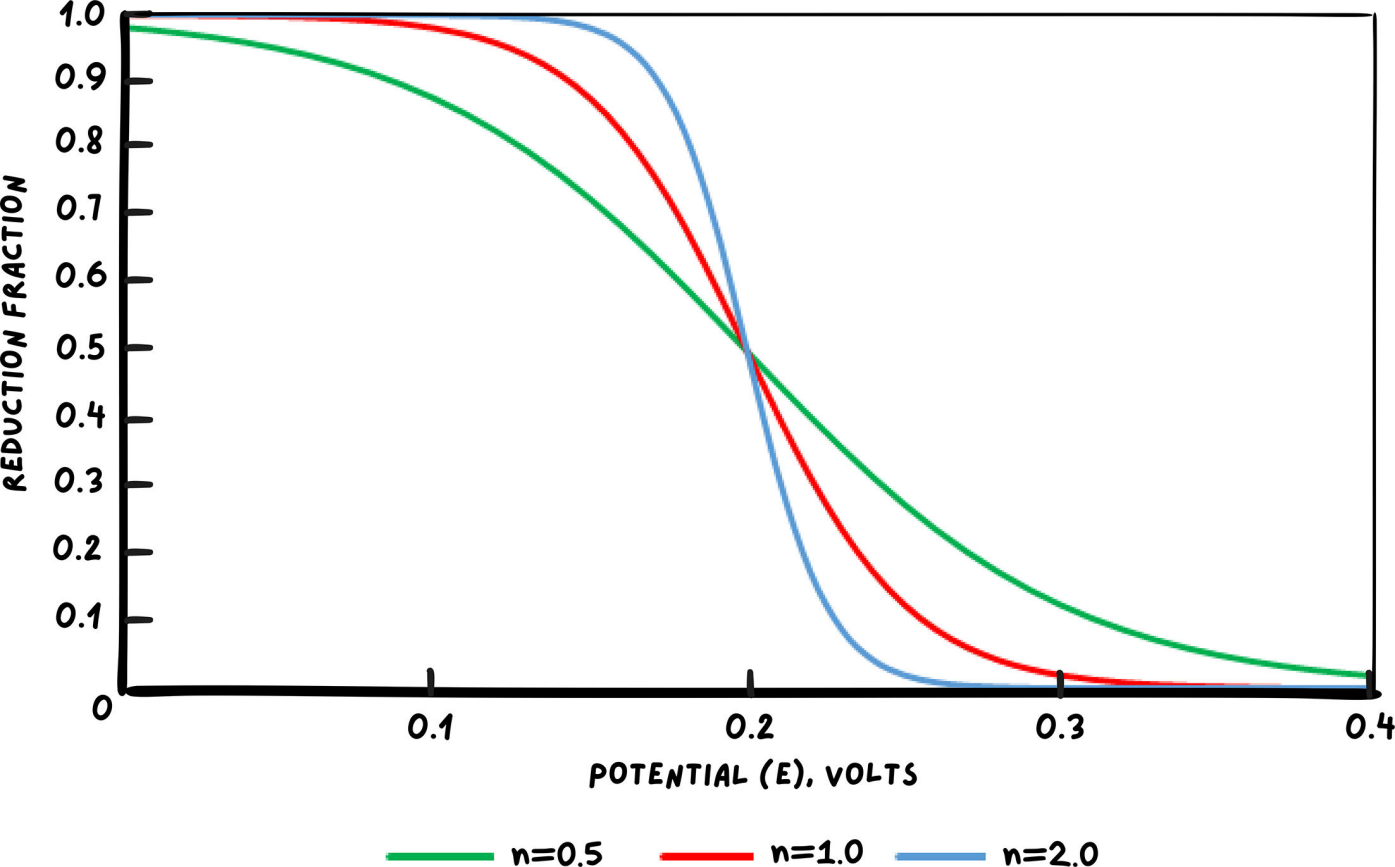
Titration curves calculated using eqn 1 for a mid-point potential of 200 mV, illustrating the effects of cooperativity on the redox behavior. The red curve was calculated considering *n* = 1 and represents a Nernst curve with no cooperativity. The blue curve was calculated considering *n* = 2 and demonstrates the effect of positive cooperativity, characterized by a steeper slope. The green curve was calculated considering *n* = 0.5 and represents the effect of negative cooperativity, where the slope is flatter than the Nernstian reference. All curves were calculated considering the same midpoint potential.

For interacting centres titrating in a similar potential range, their titration curves have a shape that is different from that defined by the Nernst curve (Figure 3). A slope flatter than the expectation from the shape of the Nernst equation reveals repulsive interactions and is a sign of *negative cooperativity*. This is what is expected from electrostatic interactions between particles of the same charge in close proximity, such as electrons. When the slope is steeper than the shape defined by the Nernst curve, it reveals attractive interactions that overcome the electrostatic repulsion. This *positive cooperativity* requires redox-linked structural alterations in the protein [12].

In both cases, these are *homotropic cooperativities* between particles of the same nature: electrons in this case. In physiologically relevant conditions, the redox potentials can be modulated further by pH, a phenomenon known as the redox-Bohr effect [13,14]. These *heterotropic cooperativites* between electron and proton binding can be parsed from the homotropic cooperativities between the redox centres by making measurements at different experimental pH conditions. To characterize a multicentre redox protein at this level of detail using a set of interdependent Nernst equations that also consider the pH dependence of the potentials is mathematically cumbersome. A more streamlined calculation can be performed by considering the free energy difference between the multiple states and deriving from there the equilibrium populations [15].

Considering Figure 2C to illustrate the multiple microscopic states in a four-centre redox protein with one coupled acid–base centre, the fully reduced and protonated condition of the protein can be defined as the reference state (G_1_ = 0) without loss of generality. The free energy of any other state can be related to that of the reference according to


(6) $$Gx=g_{i}+FE.$$

In the case of oxidation of one of the redox centres. Here, *Gx* is the arbitrary numbering of the states, *g_i_* is the free energy of oxidation of centre *i*, and *E* is the solution redox potential.

When there is oxidation of various redox centres, the interaction energy between them (*I*_*ij*_) has to be considered additionally in the calculation. For example, in the case of oxidation of two centres,


(7) $$Gx=g_{i}+g_{j}+I_{ij}+2FE,$$

where *gi* and *gj* are the energies for oxidation of centres *i* and *j* in the protein, respectively.

When there is oxidation of a redox centre and deprotonation of the acid−base group interacting with the redox centres, the energy of that state relative to the reference is


(8) $$G_{x}=\;g_{i}+\;g_{H}+\;I_{iH}+FE+2.3\;\frac{RT}{F}\;pH.$$

The resulting free energy values for any of these transitions can be related to the reduction potential using the standard thermodynamic relationship


(9) ΔG=−nFE.

The free energies of transition from the reference state can be used to determine the relative populations of the individual states using the Boltzmann equation:


(10) $$P_{x}=exp\left(-\frac{F}{RT}G_{x}\right).$$

This analysis can be expanded to describe the thermodynamic parameters of multicentre redox proteins with any arbitrary number of redox centres and include more than one acid–base centre [16,17]. However, the number of parameters necessary to characterize a system with *n* interacting centres is *n* + *n*(*n*–1)/2. This characterization requires the use of methods that can provide experimental data capable of defining all the parameters. The validation that this condition is met and that all parameters are well defined by the experimental data can be obtained from fitting the model to the data and inspecting the magnitude of the diagonal elements of a covariance matrix [18].

The microscopic thermodynamic characterization of a protein with multiple redox centres provides information on which states are populated according to the experimental conditions imposed on the system. However, to determine which states and transitions are relevant during the actual multi-electron transfer process, it is necessary to obtain kinetic information at the same level of detail.

### Kinetics of multielectron transfer in biological processes

For interprotein electron transfer to occur, the donor and the acceptor must interact, and a portion of these interactions must lead to a productive complex where an efficient electron transfer event occurs [19]. Therefore, the kinetic scheme for an electron transfer reaction between the two redox proteins involves at least three steps: (i) the complex formation between the proteins, (ii) the electron transfer event, and (iii) the dissociation of the complex. Under steady-state conditions, this scenario can be approximated by a simple collisional model, where the electron transfer process is the rate-limiting step, while the formation and dissociation of the complex occur in rapid equilibrium.


Scheme 1 $$A^{ox}+B^{red}\overset{K_{1}}{⇌}[A^{ox}-B^{red}]\overset{K_{et}}{\to }[A^{red}-B^{ox}]\overset{K_{2}}{⇌}A^{red}+B^{ox}$$

**Figure 4:**
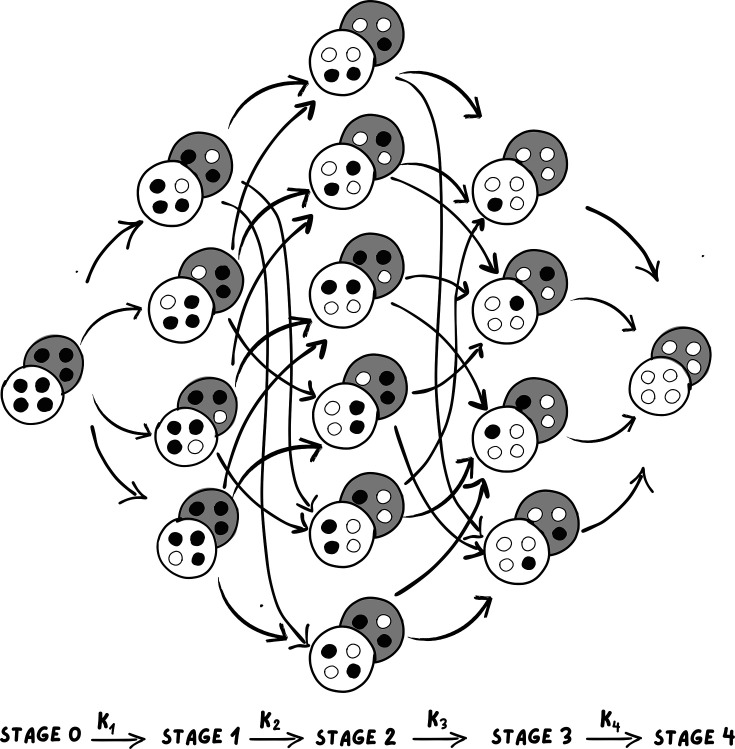
Schematic representation of the 32 microstates of a protein with four redox centres coupled to one acid–base centre, which upon reaction with an electron donor can be interconverted through 64 possible electron transfer microsteps. Black and white dots represent reduced and oxidized redox centres, respectively, while white and grey rounded squares represent deprotonated and protonated microstates, respectively.

For a redox protein with a single centre, the electron transfer rate can be easily determined using Marcus theory for electron transfer [20]. For multicentre redox proteins, the electron transfer event has to be considered for each redox centre in each particular microscopic state of the protein, which makes the characterization of the process more complex [21]. For a multicentre redox protein, it is necessary to have a detailed characterization of the thermodynamic properties of the protein to be able to fully describe the kinetic process. For example, in a protein containing four redox centres coupled to one acid–base centre, it is necessary to consider 16 protonated and 16 deprotonated microstates that are in fast protonation exchange to fully describe the system (Figure 4). Upon reaction with an electron donor or acceptor, these 32 microstates interconvert through 64 possible electron transfer microsteps, each characterized by a microscopic rate constant (k_i_^j^). The proximity of the centres in the protein makes the internal redox equilibrium within the microstates at the same oxidation stage, i.e., within columns in Figure 4, much faster than the arrival or departure of electrons from/to a redox partner encountered via diffusion. This simplifies the analysis of this system into a kinetic scheme of four consecutive macroscopic electron transfer steps (Figure 4).

The macroscopic rate constant of each consecutive electron transfer step is given by the weighted average of the microscopic rate constants of interconversion of the microstates that participate in that step, with the weights given by the relative thermodynamic equilibrium populations,


(11) $$K_{step(1-4)}=\sum_{i}^{n}\chi_{i}^{j}k_{i}^{j},$$

where χ_i_^j^ is the population fraction of the starting state. The rate constant of each microstep depends on the driving force that is given by the thermodynamic information obtained in eqns 6-10, the reorganization energy (λ), and structural factors, according to Marcus theory of electron transfer [20]. In these conditions, and assuming that the reorganization energy and the structural factors do not change during the redox transition of the protein, the rate constant of a particular microstep is given by a reference rate constant characteristic of each redox centre, k_i_^0^, multiplied by an exponential factor that accounts for the driving force associated with the electron transfer event,


(12) $$k_{i}^{j}=k_{i}^{0}\;exp\left[\frac{e_{i}^{j}F}{2RT}\left(1+\frac{e_{D}}{\lambda }-\frac{e_{i}^{j}}{2\lambda }\right)\right].$$

­In this equation, $e_{i}^{j}$ is the reduction potential of the redox centre *i* in that microstep and e_D_ is the reduction potential of the electron donor [22]. This model has been successfully applied to several MHCs *c* to investigate the kinetics of their reduction or oxidation process because the thermodynamic data necessary to define the individual microscopic redox potentials and interactions were available [23]. For example, the reduction of cytochromes *c*_3_ from different sulfate-reducing bacteria using sodium dithionite showed that there is no direct correlation between the rate constants and structural factor of the hemes (i.e., accessibility of the reducing agent, dipole moment, charges of the protein, ionic strength, or reduction potential) [23–26]. This model was also used to determine the reduction and oxidation rate constants of the individual hemes in tetraheme cytochrome STC from *Shewanella* spp. [27]. We have shown that, despite having the weakest driving force, heme I is the sole electron receiver. It also serves as the major center of electron egress, with heme IV making a smaller contribution [27]. This microscopic kinetic analysis was also extended to study the redox catalysis of the fumarate reductase flavocytochrome *c*_3_ from *Shewanella* spp. [16]. That work revealed that the different redox stages of this protein display different catalytic rates, which account for its distinct physiological functions in the bioenergetic metabolism of *Shewanella*. It was proposed that at low electron flux, flavocytochrome *c*_3_ receives electrons from CymA, and since it will not charge up, it will transfer electrons to outer membrane reductases. As the electron flux increases, flavocytochrome *c*_3_ will become more reduced, leading to a shift towards efficient catalysis of fumarate reduction [16]. Those studies demonstrated that a microscopic thermodynamic and kinetic analysis can be adapted to different multicentre proteins and redox processes, enabling the acquisition of structural and functional information about the redox centres and their significance to various electron transfer processes and redox catalysis. In parallel to multielectron redox catalysis, one aspect of biological electron transfer relevant for anaerobic environments is conductivity along micrometer distances via protein wires containing multiple, closely spaced redox centres.

### Electron transfer and conductivity

Conductivity through redox proteins involves the participation of orbitals. It can proceed by two general mechanisms depending on the rates of electron transfer through the molecule relative to the rates of nuclear relaxation induced by thermal fluctuations under the experimental conditions being probed:

— In coherent resonance tunnelling, the residence time for tunnelling electrons on the molecule is negligible compared with the timescale of nuclear motion (~10^-13^ s) [20].
— In incoherent electron hopping, electron transfer (ET) involves electronic levels of the redox centres, and rates through the molecule are similar to or slower than the timescale of nuclear motion. This provides time for spontaneous thermal relaxation of the nuclear configuration to stabilize the system upon the redox transition. In these conditions, the redox potentials of the redox centres can impact the conductivity.

Conductivity (*σ*) is related to the mobility and concentration of charge carriers, which are influenced by the rate of electron transfer. It can be expressed as


(13) σ=neμ,

where *n* is the density of charge carriers, e is the elementary charge, and *μ* is the mobility of the charge carriers.

In a simple approximation, the mobility of the charge carriers can be considered proportional to the electron transfer rate. Thus, conductivity can be expressed as a function of the rate of electron transfer according to


(14) $$\sigma \propto nek_{ET}$$

For the case of incoherent hopping, when the rate of electron transfer is not experimentally available, an estimate can be obtained by applying, for example, the simplified version of the Dutton–Moser ruler:


(15) $$\ln k_{ET}=13-0.6\left(R-3.6\right)-3.1\frac{{\left(\Delta G+\lambda \right)}^{2}}{\lambda },$$

where *R* is the distance between the redox centres, ∆*G* is the driving force and *λ* is the reorganization energy, usually taken as 1 eV for heme proteins [22].

The immediate consequence of eqn 15 is that the distance between redox centres and driving force exert opposite effects on the rate of electron transfer and, therefore, on the conductivity of the protein under consideration. One process of particular emerging interest in the conductivity of biological molecules is the phenomenon of EET. The distinguishing feature of this bioenergetic metabolism is the establishment of an electrical interface between the microbial metabolism and extracellular electron donors or acceptors. This interface involves the participation of proteins and biological structures with a great diversity of structures and physiological roles, for which structural and electron transfer data are being reported in the literature.

### Electron transfer across cell surface envelopes

**Figure 5:**
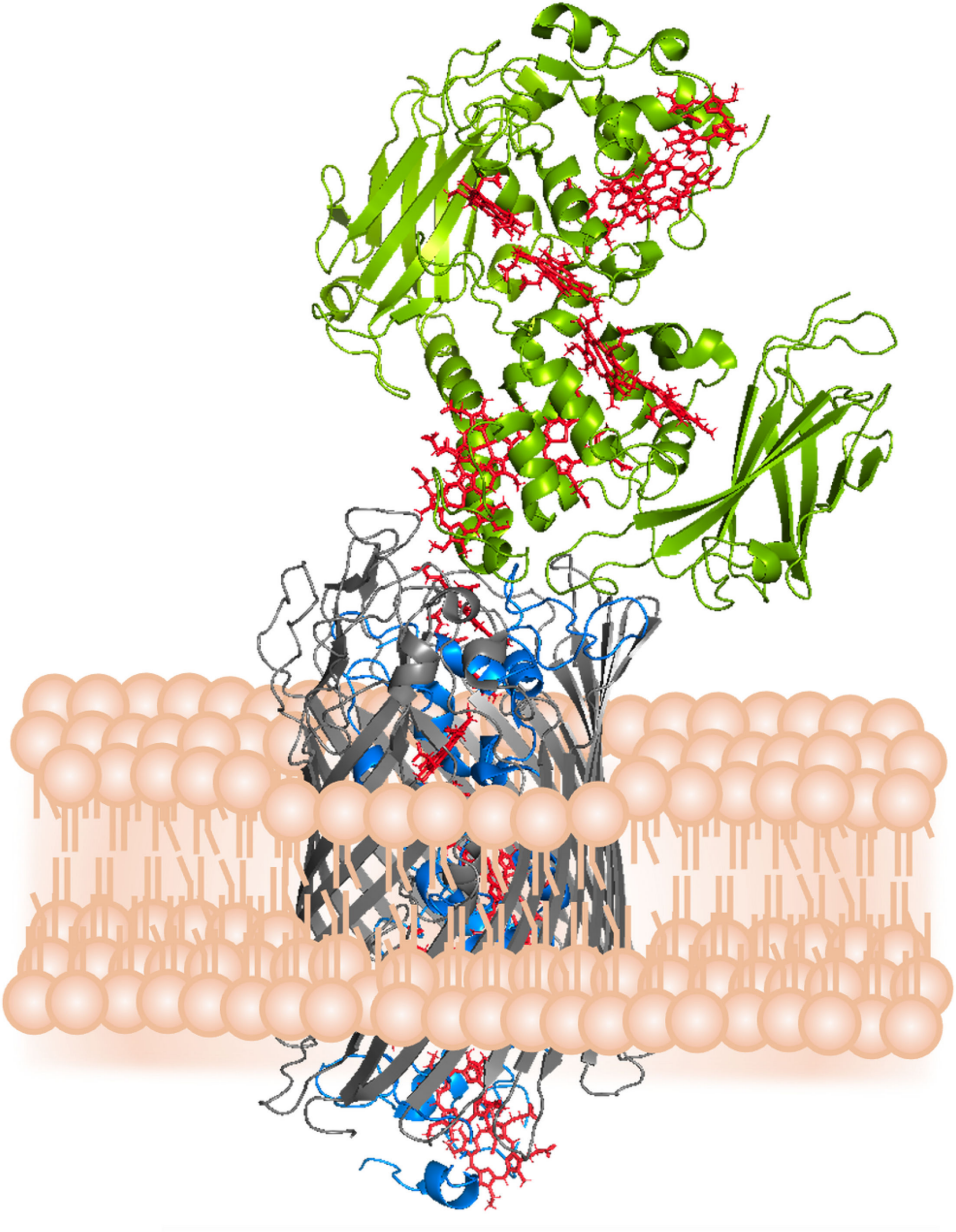
Representation of the MtrCAB structure from *S. baltica* (PDB ID: 6R2Q). The transmembrane porin MtrB (gray) accommodates the MHC MtrA (blue) and associates with extracellular cytochrome MtrC (green). Hemes are represented in red sticks.

The concept of porin–cytochrome complexes was proposed by Richardson and co-workers to explain the observation of electron transfer across cell surfaces. In Gram-negative bacteria, it is made of a β-barrel porin that extends across the outer membrane. This porin accommodates a MHC in its cavity to create a pathway for direct electron transfer across the outer membrane [28]. Metal-reducing bacteria such as *Geobacter sulfurreducens* and *Shewanella oneidensis* MR-1 use these complexes to transfer electrons to insoluble extracellular acceptors, whereas metal-oxidizing bacteria such as *Sideroxydans lithotrophicus* ES-1 and *Rhodopseudomonas palustris* TIE-1 use porin–cytochrome complexes to uptake electrons from insoluble extracellular donors [29]. The porin–cytochrome complex may be complemented by additional proteins located at the surface of the cell. This was proposed originally when the structure of the decaheme cytochrome MtrF was reported [30] and was later experimentally observed in the MtrCAB complex from *Shewanella baltica* (Figure 5) [31]. The structurally characterized MtrCAB complex contains a total of 20 bis-histidine co-ordinated *c*-type hemes with a total chain length of 185 Å, which includes the 80 Å long cytochrome MtrA [31]. MtrA is oriented in the porin so that heme 1 (numbered according to the position of the binding motif in the polypeptide chain) faces the periplasm and heme 10 faces the cell surface.

The hemes of MtrA form a conductive wire with edge-to-edge distances between the hemes closer than 7 Å. Heme 10 of MtrA displays an edge–edge distance of 8 Å to heme 5 of MtrC, which ensures fast electron transfer between the two proteins [31,32]. The topological arrangement of hemes in the MtrC forms a so-called staggered cross that spans two domains of the protein (domains II and IV). In these domains, neighboring bis-histidine co-ordinated hemes display edge-to-edge distances closer than 7 Å, which ensures fast intramolecular electron transfer. Indeed, an intrinsic electron transfer rate on the order of 10^9^ s^-1^ was experimentally determined between the hemes of MtrC from *S. oneidensis* MR-1 [33]. Heme 10 is on the opposite side of the protein to heme 5 and is the likely electron egress site via interactions with metal oxides or electrodes [31,34]. This is in agreement with the identification of a nearby hematite-binding motif, Thr–Pro–Ser/Thr [35]. Additionally, MtrC displays two flavin binding domains, designated I and III in the structure, which suggest a more complex role in EET than simply serving as electron wires [36]. Both MtrA and MtrC display pH-dependent redox potentials in a wide range that encompasses the physiological conditions. MtrA from *S. oneidensis* MR-1 is redox active between −400 and −100 mV versus the standard hydrogen electrode (SHE) at pH 7 [37], whereas MtrC titrates in the range of −400 to +50 mV at pH 7 [38]. MtrC associates transiently with another decaheme cytochrome on the surface of the outer membrane of *Shewanella* spp. called OmcA, which has a similar topological organization of the heme core as MtrC and also has a hematite binding site near heme 10 [35,39]. OmcA is redox active between −400 and 0 mV at pH 7.6 and also displays the redox-Bohr effect [9]. In the case of this protein, there is experimental small-angle X-ray scattering (SAXS) data showing a significant conformational change upon reduction, with contraction of the protein dimensions [40].

*Shewanella* spp. co-opted the porin–cytochrome arrangement for the reduction in diverse extracellular electron acceptors. MtrDEF appears to be organized in the same way as MtrCAB, and the structure of MtrF in the oxidized state was used to perform theoretical calculations of the individual heme potentials, considering all hemes oxidized and all hemes reduced [41]. This corresponds to the transitions from stage 10 to 9 and from stage 1 to 0 in the nomenclature of Figure 2. These calculations showed that the individual potentials of the hemes in the oxidized MtrF range from −336 to −47 mV, whereas for the reduced MtrF, they range from −392 to −176 [41]. The range of the individual values broadly agrees with the experimentally determined range of redox activity of the protein that spans from −400 to +100 mV [30]. The calculated potentials of the individual hemes were found to favour downhill electron transfer from domain IV to II in the oxidized state and to make it more favourable in the reduced state to reduce hemes 2 and 7 that are proposed to be near the flavin binding sites [36].

MtrC and OmcA react with Fe(III) complexes with biphasic kinetics with rates that correlate with the driving force of the reaction considering the potentials of each complex [42]. These decaheme cytochromes, together with MtrF and a homologue with an additional heme called UndA, are also capable of reducing soluble redox mediators of different electrostatic nature, such as flavin mononucleotide (FMN) and anthraquinone-2,6-disulphonate (AQDS), which are negatively charged; riboflavin (RF), which is uncharged; and phenazine methosulfate (PMS), which is positively charged. The extent and rate of reduction in these compounds again correlate with driving force as given by the relative potential of the shuttles vs. the redox-active envelope of the cytochromes, with the less negative MtrF being distinctly slower [36].

Rates of electron transfer by these MHCs reported in the literature vary by orders of magnitude, with different experimental setups giving rise to different values that cannot be easily compared. For example, initial rates of electron transfer from reduced methyl viologen to Fe(III) oxide nanoparticles through the MtrCAB complex encapsulated inside proteoliposomes were reported in the range of 1.1 × 10^3^ to 8.5 × 10^3^ e.s^−1^ [43]. The interfacial electron transfer kinetics between the cytochromes MtrF and MtrC with the pyrolytic graphite edge electrode were determined by fitting the experimental data obtained at pH 7.0 from protein film voltammetry (PFV) to the Butler–Volmer model. The resulting rates are 220 s^-1^ for MtrF at 20°C and 276 s^-1^ for MtrC at 10°C, respectively (Table 1) [9,30]. Electron transfer from OmcA to hematite was measured by spectroscopy and estimated to occur at an electron flux of about 10^13^ electrons/cm^2^/s for a surface coverage of 10^14^ molecules/cm^2^ [50]. Additionally, single-molecule conduction studies of OmcA and MtrC suggest that different mechanisms operate in the two proteins. OmcA appears to conduct electrons via a coherent tunnelling mechanism that does not involve the participation of heme-centred electronic states. In contrast, MtrC shows two conductance peaks in the tunnelling spectra that are consistent with orbital-mediated tunnelling via heme centres and match the experimental redox potential envelope of the protein [51]. These differences in the conduction mechanism correlate with the different physiological roles proposed for these proteins. For example, in a study with goethite, OmcA has shown a major role in enhanced affinity and rapid attachment, whereas MtrC increased the contact area and promoted interfacial reaction [52].

**Table 1:** Overview of electron transfer characteristics of MHC exposed on the bacterial surface

Surface exposed MHC	PDB and gene ID	Microorganism	No. of hemes	Titration range (mV versus SHE)	Rate of electron transfer (s^−1^) to an electrode (pyrolytic graphite edge)	Rate of electron transfer (s^–1^) to hematite	References
MtrF	3PMQ SO_1780	*Shewanella oneidensis* MR-1	10	−400 to +100	220	nd	[30,31]
UndA	3UCP SO_1788	*Shewanella sp.* HRCR-6	11	nd	nd	nd	[44]
OmcA	4LMH SO_1779	*Shewanella oneidensis* MR-1	10	-400 to 0	nd	0.11	[34,45,46]
MtrC	4LM8 SO_1778	*Shewanella oneidensis* MR-1	10	−400 to +50	276	0.26	[9, 38, 45, 47]
OcwA	6I5B TherJR_2595	*Thermincola potens* JR	9	-450 to +100	150	nd	[48]
OmhA	6QVM	*Carboxydothermus ferrireducens*	11	-400 to +150	nd	nd	[49]

In addition to MtrAB and MtrDE involved in iron mineral reduction, another porin–cytochrome pair located in the outer membrane of *Shewanella* is the DmsEF pair. DmsEF associates with outer membrane proteins DmsB and DmsA to reduce dimethyl sulfoxide (DMSO) under anaerobic conditions [53]. The electrochemical active range of potentials of DmsE is similar to that of MtrA (Table 2) and displays the redox-Bohr effect only at low pH [54]. The interfacial electron transfer rates of DmsE and MtrA to a pyrolytic graphite edge electrode were determined using the Butler–Volmer model to be ~122 s^-1^ [54] and 126 s^-1^, respectively [9].

**Table 2:** Electron transfer properties of MHCs embedded in the cell surface reported in the literature.

Microorganism	MHC embedded in the cell surface	No. of hemes	Titration range (mV versus SHE) and references
*Shewanella oneidensis* MR-1	MtrA (SO_1777)	10	−400 to −100 [37]
	DmsE (SO_1427)	10	−360 to −90 [54]
*Rhodopseudomonas palustris* TIE-1	PioA (Rpal_0817)	10	−400 to +250 [55]
*Sideroxydans lithotrophicus* ES‑1	MtoA (Slit_2497)	10	−400 to +100 [56]

Porin–cytochrome pairs also operate in the reverse direction. The Gram-negative bacterium *R. palustris* TIE-1 collects electrons from reduced iron minerals in the environment to drive photoferroautotrophic metabolism [57]. This is performed by the decaheme cytochrome PioA (a MtrA homologue), which is sheathed by the outer-membrane porin protein PioB (an MtrB homologue) [58]. Unlike MtrAB, the structure of the PioAB complex of *R. palustris* TIE-1 has not yet been experimentally determined. PioA titrates in the range between –400 and +250 mV vs. SHE at pH 7.4, with minimal variations in the redox properties between pH 6.4 and 9.4 [55]. Another Gram-negative bacterium, *S. lithotrophicus* ES-1, also utilizes a MtrAB homolog designated MtoAB to uptake electrons from ferrous iron and transfer them to periplasmic acceptors [56]. Titration of MtoA at pH 7.1 was modelled considering ten individual independent potentials in the range of −310 to +30 mV vs. SHE, which is pH dependent in the range of 7.1–9.2. On the cell surface, the interaction of MtoA with different ferrous iron ligands was measured using stopped-flow spectrophotometry, showing the following order of reaction rates from highest to lowest: Fe(II)Cl_2_, Fe(II)-citrate, Fe(II)-NTA, and Fe(II)-EDTA [56].

The thick peptidoglycan cell wall (20–80 nm) of Gram-positive bacteria appears to be able to stabilize embedded MHC involved in EET without the need for a porin [59,60]. *Thermincola* spp. generates current in microbial fuel cells by utilizing MHC for direct electron transfer [59–61]. CwcA (Tfer_0075) is a cytochrome proposed to be embedded in the cell wall, where it can form a stacked arrangement to create a pathway for electron transfer to the extracellular environment. The structural model of CwcA, based on the experimental structure of cytochrome OmcS from *G. sulfurreducens*, shows six hemes packed 3.5–6 Å apart, where heme 5 has an open co-ordination position on the distal side of the heme that is suitable for binding to a histidine from a subsequent subunit [59]. CwcA and its homologue in *Thermincola potens* JR are proposed to attach to terminal reductases on the surface of the cell wall [59,60]. The crystal structures of two terminal reductases (OcwA and OmhA) from Gram-positive thermophilic bacteria are reported in the literature [48,49]. These structures revealed a close similarity in protein folding and heme configuration to MHCs involved in nitrogen and sulfur biogeochemical processes [62]. The nonaheme cytochrome OcwA shows a redox potential window that ranges from −450 to +100 mV vs. SHE. The potentials are pH-dependent between pH 5.5 and 8.4 and drift to more negative values at higher pH, as expected from the redox-Bohr effect. The interfacial ET rate to the pyrolytic graphite edge electrode is approximately 150 s^-1^ [48]. The undecaheme cytochrome *c* OmhA from *Carboxydothermus ferrireducens* displays a redox-active potential window of −400 to +150 mV vs. SHE at pH 7.5 [49]. Both proteins also display putative hematite-binding motifs in their sequences ,which supports the assignment of their physiological function.

### Nanowires

Electroactive prokaryotes can also produce extracellular appendages called generically nanowires to facilitate long-range EET [63–65]. These biological nanowires can be classified into three different types: MHCs [63,66], electrical pili (e-pili) [64] and outer membrane extensions [67]. While outer membrane extensions are well accepted as a nanowiring mechanism for EET in the electroactive model organism *S. oneidensis* MR-1, the role of MHC and e-pili nanowires in *G. sulfurreducens* PCA is still the subject of an intense dispute in the literature [68–72].

**Figure 6:**
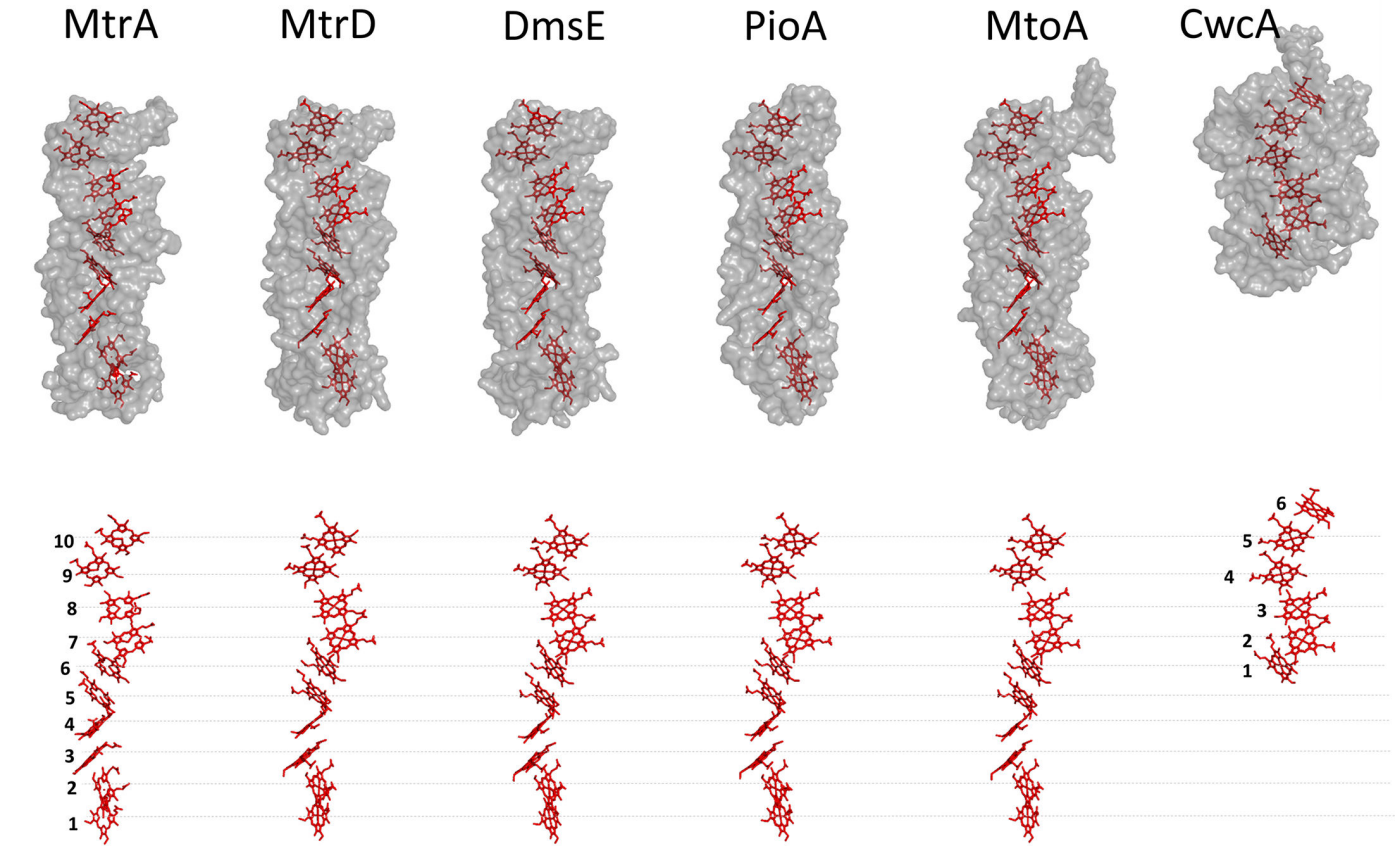
Three-dimensional structures of the porin–cytochromes predicted by AlphaFold 2: MtrD: AF-Q8e.g.30; DmsE: AF-Q8E9C4; PioA: AF-A1EBT2; MtoA: AF-D5CMQ0; and CwcA: AF-A0A0L6W663. The hemes were inserted into the structures as described in the literature [76]. For MtrA, the experimentally reported structure was used (PDB code: 6R2Q_A).

Five polymerizing MHC nanowires displayed at the surface of the cell were discovered and characterized structurally from bacteria and archaea: OmcE, OmcS and OmcZ from *G. sulfurreducens*; AvECN from *Archaeoglobus veneficus,*; and PcECN from *Pyrobaculum calidifontis*. OmcS is a hexaheme cytochrome that was previously thought to associate with the pilin protein PilA [73]. The structural characterization showed that OmcS is a nanowire, forming conductive micrometre-long polymers through inter-subunit heme coordination. Heme 5 of one subunit is co-ordinated by a histidine of the adjacent subunit. Edge-to-edge distances between hemes range between 3.4 Å and 6.1 Å, which allow fast electron transfer by multistep hopping,and the conductivity of OmcS was determined to be on average 24 mS cm^-1^ [74]. The redox-active range of this protein spans from −310 to 50 mV [75]. The tetraheme cytochrome OmcE is phylogenetically unrelated to OmcS but is structurally analogous, as shown by the good superposition of the four hemes in OmcE with hemes 3–6 of OmcS (Figure 6). This structural similarity extends to the interaction of adjacent monomers, with heme 2 of OmcE also co-ordinated by a histidine from an adjacent subunit. The edge-to-edge distances between hemes are 3.8–6 Å. The nanowires formed by the tetraheme cytochromes AvECN and PcECN are unrelated to the previous MHC nanowires but preserve the highly analogous heme arrangements to OmcS and OmcE, with edge-to-edge distances in the range of 4.3–6.6 Å and 4.2–6.4 Å, respectively. These nanowires present features of elevated protein stability that, in the case of AvECN, include a stacking arrangement of the subunits where three of the four hemes are co-ordinated on the distal side by histidines from another subunit [77]. OmcZ is an octaheme cytochrome that has all hemes co-ordinated by two histidines. The redox active range of this protein spans from −420 to −60 mV [78]. Unlike OmcS, OmcZ filaments show ramification in a web-like structure [66,79]. This may be the reason for OmcZ having the highest conductivity ever reported for a cytochrome nanowire even though hemes display a similar range of edge-to-edge distances between 3.6 Å and 5.7 Å. The conductivity of OmcZ, reported to average 27.5 S cm^-1^, is approximately 1000-fold higher than OmcS in air-dried conditions [74,80].

**Figure 7:**
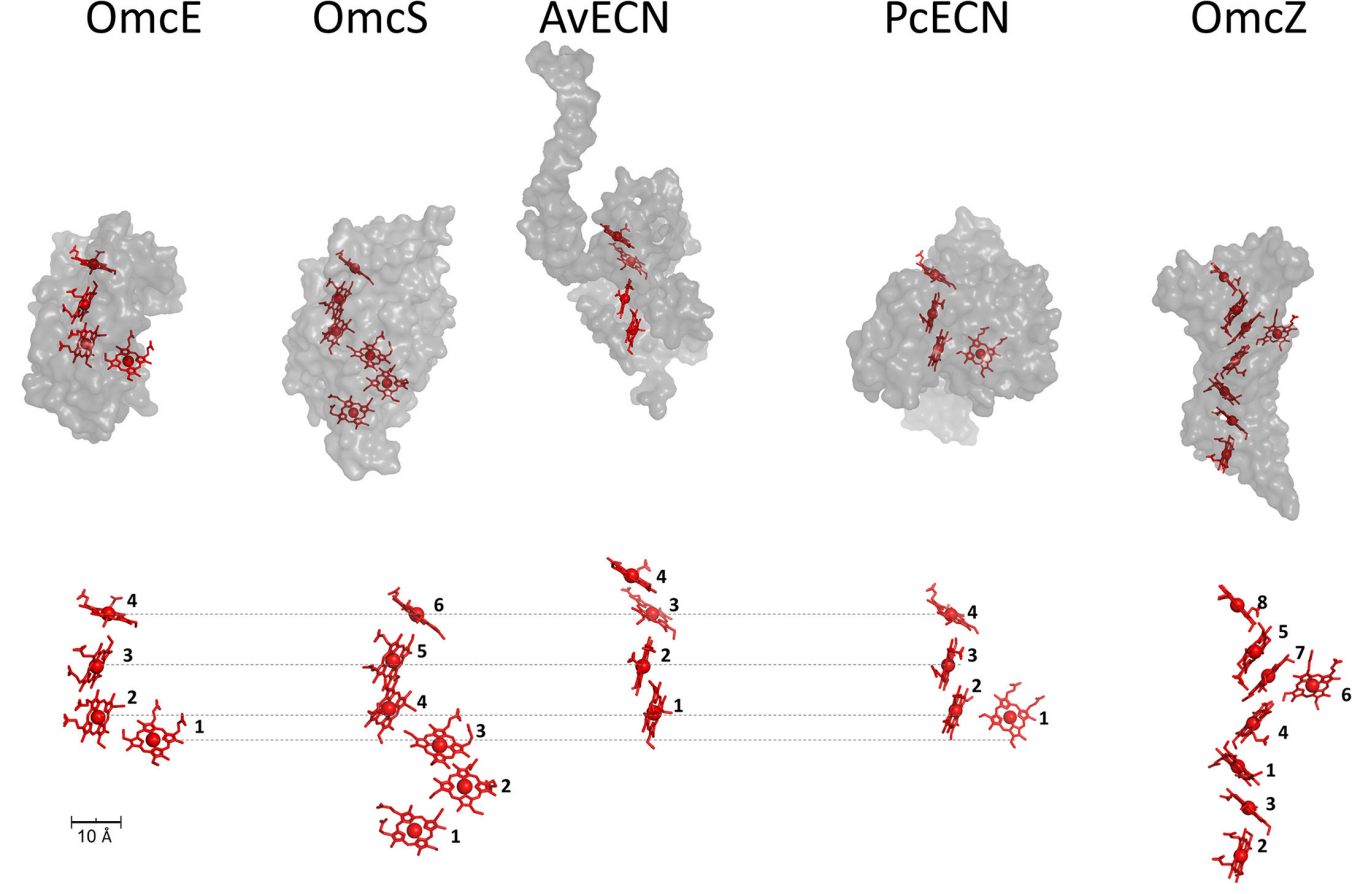
Three-dimensional structures of the polymerising MHC nanowires. Hemes are numbered according to the order of attachment to the polypeptide chain. Portions of heme arrangement similarity are present in all except OmcZ, and dashed lines connect hemes with similar arrangements. PDB codes used: 7TFS_A (OmcE), 6EF8_A (OmcS), 7LQ5_A (OmcZ), 8E5G_A (AvECN), and 8E5F (PcECN). The scale bar is represented at the bottom left corner.

Type IV pili (T4p) are protein filaments that are composed of repetitive pilin monomers and have diversified roles in prokaryotes, including motility, adhesion, biofilm formation and trafficking of molecules [81]. The proposal of a role as a terminal reductase in EET was a paradigm shift on both T4ps and EET [64], and after almost two decades, it is still controversial. *G. sulfurreducens* e-pili also known as PilA is a type IVa pilin that does not contain the globular domain of the C-terminal of classical T4p. E-pili were shown to be essential for EET [64,72]. Furthermore, *Pseudomonas aeruginosa* electroactivity was greatly increased by heterologous expression of *G. sulfurreducens* e-pili, with a 20-fold resistance reduction in comparison with wild-type *P. aeruginosa* [82]. The proposed mechanism for long-range electron transfer is dependent on the presence of aromatic amino acids closely packed to allow for overlapping π-orbitals, emulating a metal-like conductivity [82–85]. Yet, the reports on e-pili conductivity are contradictory [[64,68,86,87]], ranging at physiological conditions from 1 mS cm^−1^ [68] to 4 S cm^−1^ [87,88]. Alternatively, the role of e-pili in EET has been proposed to be the promotion of secretion of OmcS and OmcZ [68].

*S. oneidensis* MR-1 has an additional nanowiring strategy. In this case, these nanowires are outer membrane and periplasmic extensions [67] and outer membrane vesicles [89] that are also conductive and transport electrons through micrometer-long distances [65,89]. OmcA and MtrC were shown to be up-regulated and essential at the cell surface for this nanowiring mechanism [67,90]. The conductivity mechanism for *Shewanella* nanowires was found to involve discrete energy levels with a higher density of states attributed to the molecular components of the nanowire and allows for rates of up to 10^11^ s^-1^, which favour a coherent band mechanism over incoherent hopping [91]. Computational simulations comparing the MtrCAB complex vs. OmcS predict that the *Shewanella* complex has one order of magnitude slower electron transfer rate [92]. The measured conductivities of *S. oneidensis* MR-1 nanowires are within the range of 1 µS cm^−1^ [65], which is substantially lower than the protein nanowires discussed above.

### Cable bacteria skeletons

One of the main discoveries about electron transfer in biological systems took place in 2010 with the description of filamentous bacteria capable of transferring electrons over centimetre-long distances [92,93]. These unique living structures, known as cable bacteria, transfer electrons along multicellular and non-branched filaments where the electrical current crosses through the different natural redox gradients formed in marine and freshwater sediment environments [94].

Cable bacteria owe their name to the presence of longitudinally parallel conductive fibres that are embedded in the periplasm and extend to the outside of the cell. These fibres are interconnected at the cell-to-cell junction [95]. They are resistant and hard to shed and are considered to remain mostly intact structurally and functionally after the harsh extraction process used to generate the so-called cable bacteria skeletons (CBSs) [96,97]. The proposed structure for the periplasmic fibres consists of an electrically insulating protein outer layer with a very conductive Ni-S-rich protein central core [98].

Reduction potentials have not been reported for CBS, but chemically reduced fibres were found to be twice as conductive as oxidised ones, implicating the redox state of the proposed sulfur-ligated Ni-dependent cofactor in the electric conductivity mechanism of cable bacteria [98]. The literature on the conductivity of cable bacteria fibres and skeletons shows a great dispersion of values. For example, a recent publication reports a median value of 27 S cm^-1^ with measurements spread between 2 and 564 S cm^-1^ [99]. Even this median value is significantly higher than the value of 1 μS cm^-1^ reported for the *Shewanella* cytochrome nanowires or the 0.02–0.04 S cm^-1^ reported for the *Geobacter* OmcS type nanowires, but comparable to the values of 4–30 S cm^−1^ obtained for the *Geobacter* OmcZ type nanowires at pH 7 [63,65,74]. The mechanism of conduction in CBS is still unknown, but mathematical modelling of the conduction properties showed that electron transfer in cable bacteria fibres is compatible with a hopping mechanism between sites [100,101].

## Conclusion

Multielectron transfer can be approached at different levels of detail, depending on the discrimination capacity of the methods available and the specific question being investigated. Interest in this process can stem from research on electron transfer, multielectron catalysis, or long-range conductivity. The latter is of great biological relevance, which we illustrated briefly in the context of EET that establishes the electrical contact between microbial metabolism and extracellular conductive minerals or electrical circuits. However, the molecular mechanisms underpinning conductivity in EET remain to be clarified. Table 3 shows several aspects of conductivity in the context of EET that are ripe for further exploration.

**Table 3:** Values reported in the literature for the conductivity of diverse molecular structures that participate in EET.

Conductor	Conductivity (mS/cm)	Reference
Dry ferrocytochrome *c*_3_ DvM	17–125	[102]
Dry ferricytochrome *c*_3_ DvM	0.000001	[102]
OmcZ	27,500	[80]
OmcS	20–40	[74]
Periplasmic extension	0.001	[65]
e-pili	1–4300	[68,87,88]
Cable bacteria skeleton	2000–564,000	[99]

The first is that the work on cytochrome *c*_3_ from *Desulfovibrio vulgaris* Miyazaki strongly suggests that the conductivity mechanism may be dependent on the redox poise of the hemes, at least when the measurement is done in dry conditions. The second is that even when considering only outer membrane cytochromes OmcZ and OmcS, conductivity spans three orders of magnitude. However, the difference in the values of conductivity reported is difficult to reconcile with what is known about the similar distances between hemes in their structure and about the similar redox-active envelope of these proteins [103]. This observation hints at different mechanisms of conductivity in these two proteins that remain to be proposed, but that may relate to the observation that MtrC and OmcA have different conduction mechanisms despite sharing similar structures [51]. The third is that when multiple measurements are reported for the same molecular structure, the range of values is spread by up to three orders of magnitude, which may relate to the controversy of the actual nature, purity and homogeneity of the preparations [68,87,98,99]. The dispersion of values reported in the literature makes it difficult to establish firm proposals on the molecular mechanisms of conduction. Conduction by multistep hopping in MHCs *c* appears more intuitive given the organization of the hemes in the structure with short edge-to-edge distances and regular patterns of organization of hemes as parallel pairs and ‘T-junctions’ as illustrated in Figures 6 and 7 and noted in the literature [104]. However, evidence exists in support of coherent band conduction via electronic states delocalized across the proteins [105,106]. Indeed, nature may make use of the two mechanisms depending on the degree of orbital overlap between hemes in the structure [107]. The two mechanisms can actually be distinguished on the basis of the temperature dependence [80,102,108,109]. Going full circle to the beginning of this paper, the possibility of band conduction being relevant in biological electron transfer was actually proposed originally by Szent-Györgyi, and the methods and data reviewed here show that it is now possible to explore if and in what circumstances it occurs [110].

## Abbreviations

AQDS: anthraquinone-2,6-disulphonate
CBSs: cable bacteria skeletons
DMSO: dimethyl sulfoxide
EET: extracellular electron transfer
ET: electron transfer
FMN: flavin mononucleotide
HiPIPs: high-potential iron–sulfur proteins
MHC: multiheme cytochrome
PFV: protein film voltammetry
PMS: phenazine methosulfate
RF: riboflavin
SAXS: small-angle X-ray scattering
SHE: standard hydrogen electrode
T4p: type IV pili
